# Evaluation of Common Factors of Periodontitis and Cardiovascular Disease in Patients with the Acute Coronary Syndrome

**DOI:** 10.3390/ijerph19138139

**Published:** 2022-07-02

**Authors:** Elżbieta Dembowska, Aleksandra Jaroń, Ewa Gabrysz-Trybek, Joanna Bladowska, Grzegorz Trybek

**Affiliations:** 1 Departament of Periodontology, Pomeranian Medical University in Szczecin, 70-111 Szczecin, Poland; elzbieta.dembowska@pum.edu.pl or; 2 Specjalistyczne Centrum Stomatologii Elżbieta Dembowska, Bohaterów Warszawy 11b/5, 70-370 Szczecin, Poland; 3 Department of Oral Surgery, Pomeranian Medical University in Szczecin, 70-111 Szczecin, Poland; jaronola@gmail.com; 4 Indywidualna Specjalistyczna Praktyka Lekarska Ewa Gabrysz-Trybek, 70-111 Szczecin, Poland; ewa_gabrysz@wp.pl; 5 Department of General and Interventional Radiology and Neuroradiology, Wroclaw Medical University, M. Curie-Skłodowskiej 68, 50-369 Wrocław, Poland; joanna.bladowska@umed.wroc.pl

**Keywords:** periodontitis, cardiovascular disease, acute coronary syndrome, oral health

## Abstract

Periodontitis is a multifactorial disease causing inflammatory destruction of supporting structures of the dentition and eventually leading to its loss. This study was designed to evaluate common risk factors for periodontitis and acute coronary syndrome in the study population and demonstrate the systemic impact of periodontitis on the occurrence of acute coronary syndrome. A total of 160 patients (35 female and 125 male) were enrolled in the study. Considering the age range, the largest group of patients (118 patients) was between 55 and 65 years, which accounted for 73.8% of the total study population. There were 35 patients (21.9%) in the age group of 45 to 54 years, while the youngest age group of 35 to 44 years had as many as seven patients. Medical history and physical examination, including periodontal status, were performed. API, PD, CAL, and CPITN were evaluated. Common risk factors for periodontitis and acute coronary syndrome were assessed. The study assessed risk factors such as hypertension, diabetes, dyslipidemia, general health, smoking, height, weight, and hip circumference. In light of the above-described etiopathogenesis of atherosclerotic disease and its association with periodontal disease, it is important to emphasize preventing and treating periodontitis, especially in patients in the so-called high-risk group for cardiovascular disease. Dentists’ introduction of an appropriate prophylactic and therapeutic plan may constitute both primary and secondary prevention of cardiovascular diseases.

## 1. Introduction

Periodontitis is a disease of multifactorial etiology, causing inflammatory destruction of supporting structures of dentition and eventually leading to its loss [1]. Periodontitis is caused by an imbalance between infectious (microbial) factors, the host’s defense mechanisms, and individual susceptibility, usually genetically modified. The effect of periodontitis is the loss of alveolar bone and forming of a periodontal pocket in which subgingival bacterial plaque and biofilm form. As an organized bacterial structure, biofilm is difficult to access with chemotherapy and mechanotherapy [2].

Risk factors in the etiology of chronic periodontitis can be divided into two groups [3]:

(1) determinants, i.e., factors that are beyond the control of either the patient or the physician: age, gender, genetic factor, social status;
(2) proper risk factors, even if they cannot be eliminated, are subject to modification: oral bacterial flora, smoking, stress, diabetes, osteoporosis, and other general diseases. It should be emphasized that some risk factors are common to periodontitis and cardiovascular diseases [4,5,6,7,8].

Cardiovascular diseases are counted among social diseases. The study of the so-called standardized mortality, conducted at the age of 35–64, showed that in the group of 100,000 people, as many as 442 men and 149 women died due to cardiovascular diseases. This value was 62% higher compared to data from 15 years ago [9,10].

Myocardial infarction is a form of ischemic heart disease (IHD), which in addition to myocardial infarction, includes ischemia, stable angina, unstable angina, and sudden cardiac death. The essence of ischemic heart disease is the disproportion between the myocardium’s need for oxygen and energy and its ability to supply them. Cardiovascular disease is characterized by intravascular, fat-rich deposits that can induce thrombi, contributing to cardiac death [11]. The mechanism of myocardial infarction involves complete occlusion of the coronary artery lumen, leading to myocardial necrosis due to ischemia. Myocardial infarction usually occurs in the background of atherosclerosis of the coronary arteries in coronary artery disease.

Atherosclerosis is the leading cause of myocardial infarction and ischemic heart disease; it is a disease of the arteries that involves the formation of multifocal lesions in their inner and middle membranes, resulting in narrowing of the lumen and reduced elasticity of vessels [5]. The first reports on the etiopathogenesis of atherosclerosis date back to the 19th century, when Virchow presented the changes in atherosclerosis as a proliferative-degenerative process [12].

Epidemiological studies by Beck et al. and other authors have examined the association between cardiovascular disease and periodontal status [13,14,15]. There are many theories on how periodontitis may affect atherosclerotic plaque formation. In a simplified way, the impact of pathogenic bacteria from biofilm pockets can be divided into three mechanisms: bacteremia, dissemination of locally released inflammatory mediators, and initiation of autoimmune response.

Periodontitis is a constant, chronic reservoir of inflammatory mediators, in addition to cytokines, LPS, which may contribute to atherosclerosis. In addition, periodontitis-causing bacteria can penetrate the periodontal epithelial barrier and enter the bloodstream, leading to local atherogenic effects [16].

Herzberg and co-authors reported that Porphyromona gingivalis and Streptococcus sanguis, bacteria found in periodontitis, induce platelet aggregation and activation via protein expression. The aggregated proteins may play a role in atherosclerotic plaque formation and thromboembolic incidents [17].

The increasing prevalence of cardiovascular diseases and the emphasized bidirectional impact of these diseases and periodontitis prompt us to address this topic [18]. Our study aimed to evaluate common risk factors for periodontitis and acute coronary syndrome in a study population. This study was conducted on the Polish population—residents of Western Pomerania. To our knowledge, no previous studies have been conducted on this population.

## 2. Materials and Methods

The study was conducted on patients hospitalized for acute myocardial infarction in the Department of Cardiology, Medical University. A total of 160 patients (125 men and 35 women) were eligible for the study.

The criteria for inclusion in the study were: the occurrence of acute myocardial infarction, age up to 65 years, obtaining written consent for the research, and publication of results. The Bioethics Committee approved the formation of the Medical University (no KB-0012/06/12).

Exclusion criteria: acute inflammation of the respiratory tract, urinary tract, neoplastic disease, rheumatic disease, autoimmune disease, chronic liver disease, chronic kidney disease in stages 4 and 5, and a history of stroke.

Patients underwent a thorough anamnesis and physical examination;

Physical examination;

The cardiology part consisted of the completion of a cardiology form.

(A) Interview with the patient

General patient information (name, gender, date of birth, education);

Presence of risk factors (family history, hypertension, diabetes, smoking, dyslipidemia).

Weight, height, waist, and hip circumference;

BMI (body mass index) and WHR (waist hip ratio) (were calculated for each individual based on this information.

(B) Information obtained from the patient’s medical history (medical records)

The determination of risk factors for myocardial infarction and periodontitis was based on the patient’s medical records.

1. Hypertension

Hypertension is defined as a systolic blood pressure ≥140 mm Hg or diastolic blood pressure ≥90 mm Hg or the use of antihypertensive medication.

2. Diabetes mellitus

Diabetes was defined as a fasting blood glucose level of at least 126 mg/dL (7.0 mmol/L) or positive history of diabetes.

3. Dyslipidemia

Any lipid abnormality (above normal) was included: elevated total cholesterol, elevated LDL fraction, high TG, and decreased HDL fraction.

Total cholesterol, LDL, HDL, TG levels, CRP, and leukocytosis were recorded for each patient.

The type of infarction (STEMI, NSTEMI), coronary vessels involved in atherosclerotic lesions, and the extent of atherosclerotic lesions if coronary angiography was performed (lesion in 1–2 or 3–4 coronary arteries) left ventricular ejection fraction at the end of hospitalization were also determined.

(C) Periodontal part

After completing the cardiology form, the patients underwent a periodontal examination and completed a periodontal history and a physical examination.

1. Dental history

The history was asked about family history of periodontal disease and causes of tooth loss (caries, periodontal disease). The presence of general conditions affecting periodontal disease (diabetes, osteoporosis, drug-induced diseases, hormonal disorders) was also noted.

2. Periodontal examination

Periodontal examination of the patients was performed in a dental office setting under artificial lighting. One person performed all measurements using a standard diagnostic kit and a Hu-Friedy UNC15 (Uncirculated) bilateral periodontal probe. This probe is graduated in 1 mm increments on one end, and the other end corresponds to the WHO 621 (World health organization) periodontal probe. This particular side of the periodontal probe was used for the CPI (Community Periodontal Index) [19].

The following variables were assessed during the periodontal examination. (wisdom teeth were not included in the study):

The number of teeth was recorded.

Hygiene status was assessed using the API index (Approximal Plaque Index) [20]. Gingival bleeding was evaluated with the BOP (Bleeding on probing) index, and periodontal status was assessed with PD (pocket depth) and CAL (clinical attachment loss) measurements at 4 points around the tooth.

The CPITN index, according to Ainamo et al. (Community Periodontal Index of Treatment Needs), was used to determine periodontal status.

The CPI as the first component of CPITN was evaluated in individual sextants. For a sextant to be assessed, it must contain at least two fully functional teeth. Otherwise, it was excluded from the study and marked as (x). If a sextant had only one fully functional tooth, it was included in the adjacent sextant [19].

A code was entered for each sextant based on the following symptoms:

CPI = 0—healthy periodontium;

CPI = 1—the presence of bleeding;

CPI = 2—the presence of supragingival and/or subgingival calculus or overhanging filling;

CPI = 3—the presence of periodontal pockets 3.5–5.5 mm deep;

CPI = 4—the presence periodontal pockets with depth = or > 6 mm.

The highest CPI value from the tested sextants was determined in each patient.

Periodontal treatment needs (Treatment needs) were determined according to the scheme in Table 1.

To define periodontitis, the Page and Eke classification was adopted. The following were distinguished based on CAL and PD measurements: no periodontitis or mild periodontitis, moderate periodontitis, and severe periodontitis [21].

### Statistical Analysis

All continuous variables were checked for normality of distributions with the Kolmogorov–Smirnov test. The variables were described by means, standard deviations, and minimum and maximum values. Student’s *t*-test and Mann–Whitney tests checked the two groups’ statistical differences. The analysis of variance (ANOVA) or Kruskal–Wallis test was used for multiple groups.

Discontinuous variables were described by number and frequency of occurrence. Pearson’s χ2 test or Fisher’s exact test tested statistical relationships between discontinuous variables.

Spearman’s rank correlation was used to examine the correlation between discontinuous variables: ordinal and nominal (variables coded: 0/1) and continuous variables. Results were described by correlation coefficient r and probability *p*.

Statistically significant differences in all tests performed were considered those for which the probability *p* < 0.05.

Statistical analysis was performed using STATA 11 statistical software (StataCorp LLC, Lakeway Drive, College Station, Texas, USA) license number 30110532736.

## 3. Results

The study included 160 patients hospitalized for acute myocardial infarction in the Department of Cardiology, University of Medical Sciences. Patients aged between 33 and 65 years, including 35 women and 125 men, were included in the study. Considering the age range, the largest group of patients (118 patients) was between 55 and 65 years, which accounted for 73.8% of the total study population. There were 35 patients (21.9%) in the age group of 45 to 54 years, while the youngest age group of 35 to 44 years had as many as seven patients (4.4%).

### 3.1. Dental and Periodontal Status

Considering the dental status, 28 (17.50%) edentulous subjects were in the study group. A higher percentage of toothlessness was found among women, 25.71% (9 subjects), than among men, 15.20% (19 subjects). However, a higher percentage (73.60%) of subjects with more than six teeth in the male group than in the female group (45.71%). Among the subjects having up to 6 teeth, 24 subjects, including 14 males (11.20%) and 10 females (28.57%). The relationship between the number of teeth and gender is statistically significant. The older the patient was, the number of teeth was lower. We observed a worse situation regarding the number of teeth in females. However, the mean number of teeth in dentate patients was 14.96.

Periodontal pocket depths and clinical attachment loss (CAL) were measured in patients after acute myocardial infarction. The mean depth of all periodontal pockets (PD) patients was 3.06 mm. Periodontal pocket (PD) depth measurements in interdental spaces were chosen to assess the severity of periodontitis. Only five subjects out of 132 participating in the study had gingival pockets less than or equal to 3 mm in depth, including three men (2.83%) and two women (7.69%). In 55 subjects (41.67%), the depth value of periodontal pockets in the interdental spaces was between 4 and 5 mm. There were 42 males (39.62%) and 13 females (50.00%) in this group. The greatest number, 72 subjects (54.55%), had periodontal pocket depths in interproximal spaces equal to or greater than 6 mm. There were 61 men (57.55%) and 11 women (42.31%) in this group. These relationships were not statistically significant (Table 2).

The following criterion for assessing periodontal status was the clinical loss of connective tissue attachment at interproximal sites (CAL). The ranges of CAL values in the presented study corresponded to the American Academy of Periodontology (AAP) classification of periodontal diseases. Of the patients examined, only seven patients (5.3%) had CAL values in interproximal spaces less than 2 mm. This group was all male. In only 11 patients (8.33%), CAL values in the interproximal spaces ranged from 3 to 4 mm. There were two women (7.69%) and nine men (8.49%) in this group. The majority of subjects (114 subjects, 86%) had at least one site at the interfaces with clinical attachment loss of 6 mm or more. There were 90 men (84.91%) and 24 women (92.31%) in this group. These relationships were not statistically significant (Table 3).

In the subjects, periodontal status was diagnosed according to Page and Eke’s definition, based on two parameters: clinical loss of connective tissue attachment (CAL) and pocket depth (PD). This definition distinguishes three periodontal conditions: healthy periodontium or mild inflammation, moderate inflammation, and severe periodontitis. As shown in Table 4, there were 13 (9.85%) subjects with healthy or mild periodontitis, including five females (19.23%) and eight males (7.55%). Moderate periodontitis was noted in 48 subjects (36.36%) including 12 females (46.15%) and 36 males (33.96%). Severe periodontitis was diagnosed in 71 subjects (53.78%), including nine females (34.62%) and 62 males (58.49%).

After acute myocardial infarction, moderate periodontitis was statistically more common in women, while severe periodontitis was more common in men. These differences are statistically significant (*p* = 0.01672) and indicate more advanced periodontitis in males among those after myocardial infarction.

Table 5 shows the periodontal status of the study population as assessed by the CPITN index. There were no subjects with CPI code = 0, i.e., healthy periodontium, and CPI = 1, i.e., only gingival bleeding. However, there were three subjects with the highest CPI = 2 code (2.28%), 52 subjects with CPI = 3 code (39.39%), and 77 subjects with CPI = 4 code (58.33%). There were 7.69% (2 women) with CPI = 2 code, and half of the women had CPI = 3 code, slightly less because 42.31% (11 women) had CPI = 4 code. Among men, most because 62.26% (66 people) had CPI = 4 code, 36.79% (39 people) had CPI = 3 code and only one person (0.94%) had CPI = 2 code. The correlation between CPI and gender is statistically significant. According to the study, men had a more advanced form of periodontitis than women. This result confirms that the male gender is among the risk factors for periodontitis.

### 3.2. Prevalence of Risk Factors for Periodontitis and Acute Coronary Syndromes

To the question “Do you suffer from chronic stress?”—“yes”—was answered by 57.59% of the respondents (91 subjects). Among those suffering from chronic stress, a higher percentage was reported in the female group, 71.43% (25 subjects), than in the male group, 53.66% (66 subjects). This relationship is at the limit of statistical significance (*p* = 0.06054). The next question asked was: “Was there a family history of premature cardiovascular disease?” The results showed no statistically significant relationship between the family history given and gender (Table 6).

The prevalence of hypertension, diabetes mellitus, and cigarette smoking was evaluated in post-myocardial infarction patients. There was a high prevalence of hypertension, 80.63%, and a high percentage of those who had smoked cigarettes ever. The rate of females was not statistically different from the ratio of males regarding the prevalence of hypertension, diabetes, and smoking addiction.

Obesity was present in 38.8% of the subjects, overweight in 41.3%, average weight in 18.8%, and underweight in 1.3%. Categorizing weight according to BMI, no statistically significant differences were observed between men and women.

The type of atherosclerotic lesions was evaluated. In 139 subjects (86.88%), coronary RCA (right coronary artery) vessels were atherosclerotic; in 134 subjects (83.75%), LAD (left anterior descending) vessels. Atherosclerotic lesions in Cx (circumflex artery) vessels occurred in 119 patients (74.38%). The left coronary artery LM (left coronary) trunk was involved in 42 patients (26.25%).

As shown in Table 7, the percentage of post-MI patients with elevated CRP was high at 58.23%. The rate of subjects with elevated leukocyte count was also increased by 64.43%. The differences between CRP and leukocyte count by gender were not statistically significant.

There was a high percentage of dyslipidemia in post-infarction patients, 79.24%, with high total cholesterol (55.35%), high LDL (50.95%), and triglycerides (46.45%). However, there were no statistical differences due to dyslipidemia, TC, LDL, HDL, and TG levels by gender.

Based on WHR, we identified the type of obesity:

Gynoid, buttock-thigh obesity (“pear” type) was found in 48 subjects (30.2%).

Androidal abdominal obesity (“apple” type) was found in 111 subjects (69.8%).

The android figure was more common in men after acute myocardial infarction: 78 subjects (62.90%) than the gynoid figure: 46 subjects (37.10%). In 33 (94.29%), that is the majority of women after acute myocardial infarction; the android body type was present. Gynoid type was present in only two women after myocardial infarction (5.71%). The correlations between WHR and gender were statistically significant (*p* = 0.00036).

Table 8 shows the relationship between hypertension and the number of teeth. Hypertension was present in 60.71%, i.e., 17 edentulous subjects in 91.67% (23 subjects) with several teeth from 1 to 6 and 83.33% (90 subjects) with a number of teeth above 6. In contrast, dyslipidemia was present in 66.67% (18 subjects) of edentulous subjects in 95.83% (23 subjects) with the number of teeth from 1 to 6 and in 78.70% (85 subjects) with the number of teeth above 6. The associations between the number of teeth and the occurrence of hypertension and dyslipidemia in subjects after acute myocardial infarction are statistically significant. Hypertension and dyslipidemia were most common in the group of subjects with 1 to 6 teeth and least common in the group of subjects without teeth. On the other hand, there was no statistical relationship between the number of teeth and the incidence of diabetes or between the number of teeth and body type.

The mean number of teeth in hypertensive patients (14.25) was statistically significantly lower than in patients with normal blood pressure (18.95). However, gavage bleeding was statistically more frequent in hypertensive patients. The mean BOP in patients with hypertension was 0.43, and those without hypertension was 0.32 (Table 9).

In the group of 28 edentulous subjects, there were 16 subjects (59.26%) with normal total cholesterol and 11 subjects (40.74%) with total cholesterol exceeding the norm. In the group of subjects with 1 to 6 teeth, there were seven subjects (29.17%) with normal total cholesterol and 17 subjects (70.83%) with total cholesterol exceeding the norm. In the group with more than six teeth, there were 48 subjects (44.44%) with total cholesterol values up to 200 mg/dL and 60 subjects (55.56%) with total cholesterol values ≥200 mg/dL.

Table 10 shows a statistically significant relationship between the number of altered atherosclerotic arteries and the mean CAL value at interproximal sites. Subjects with 1–2 atherosclerotic altered coronary arteries had a statistically lower mean CAL value from interproximal sites, which was 5.95 mm, than subjects with 3–4 atherosclerotic altered coronary arteries, who had a mean value of 6.91 mm (*p* = 0.0390). This result indicates that subjects with more advanced periodontitis have a higher number of myxomatically affected coronary arterial vessels.

## 4. Discussion

In the present study, common risk factors for acute coronary syndromes and common factors in the etiopathogenesis of periodontitis and myocardial infarction were evaluated in the patients studied.

Risk factors common in the development of periodontitis and cardiovascular disease include age, gender, education, socioeconomic status, smoking, metabolic syndrome, and exposure to chronic stress. Recent publications show that there is a causal relationship between these conditions and the so-called inflammatory markers (CRP, elevated leukocyte levels, IL-6) and metabolic parameters (elevated triglycerides, cholesterol) [3,18].

Gram-negative bacteria can enter the bloodstream from the pathogenic periodontal pocket plaque by penetrating the inflammation-damaged epithelial attachment. Patients diagnosed with periodontitis are at risk of long-term bacteremia, and its occurrence depends on the severity of the inflammatory process in the periodontium [22]. Bacteria spreading with blood can directly affect the vascular endothelium.

It has been found that Porphyromonas gingivalis and Streptococcus sanguis bacteria possess PAAP (platelet aggregation associated protein) receptors on their surface, structurally similar to collagen, inducing platelet aggregation and formation of microthrombi during bacteremia, which after enlargement transform into wall thrombi, growing over the endothelium and turning into characteristic atherosclerotic plaques [23,24]. This relationship was demonstrated in their study by Sharma et al. [25].

The first report on the association between obesity and periodontal disease appeared in 1977 [26]. Since then, there have been many reports on this topic. In 2005, Vecchia et al. evaluated the relationship between overweight, obesity, and periodontitis and found that obesity was significantly associated with periodontal disease in adults [27]. In their study, Genco et al. concluded that obesity is associated with high plasma TNF-α levels, which may lead to inflammation that increases the risk of periodontal disease. However, this relationship is bidirectional, meaning that periodontal inflammation may also be involved in obesity [28]. A positive correlation between obesity and periodontal disease was also shown in a study of men aged 60 to 70 years, conducted by Linden et al. [29].

On the other hand, a study conducted by Eun-Jin et al. showed no correlation between BMI and periodontal disease [30]. However, a positive correlation was observed between central obesity and periodontitis. The study presented by Merit et al. also showed that deep periodontal pockets promote hypertension and lipid disorders [31].

In addition to myocardial infarction and periodontal disease, another civilization disease of the 21st century is stress. According to our study, severe periodontitis was more common in post-myocardial infarction patients exposed to chronic stress. The correlation between stress and periodontitis occurrence is confirmed by other scientific reports [32,33,34]. Olszewska-Czyż et al. found a positive correlation between stress and some periodontitis indices (API, approximal plaque index; SBI, sulcus bleeding index) and concluded that the level of stress index might affect the severity of periodontitis [35]. Yamakoshi et al. observed that during prolonged stress, saliva secretion is reduced, and salivary flow is slowed, resulting in increased plaque and calculus accumulation, leading to the formation of local periodontal inflammation triggers [36]. Other studies have suggested that stress may be involved in the increased secretion of interleukin-6 [37,38].

In our study, we evaluated how the number of retained teeth correlates with infarction incidence. A comparison of the mean number of teeth per patient after acute myocardial infarction and epidemiological studies is presented. The mean number of teeth in our study was 14.96 teeth. A group of 82 patients after myocardial infarction with a mean age of 56.5 years was studied by Wójcicka-Rubin et al. The mean number of teeth in the study group was 12.88 [39]. Although the mean age in the study group of Wójcicka-Rubin et al. is lower than in the patients in their study, they noted a lower mean number of teeth per patient.

The existence of a relationship between the number of retained teeth, the progression of atherosclerosis, and the risk of myocardial infarction was shown in the ICARAS study (Inflammation and Carotid Artery Risk for Atherosclerosis Study). Desvarieux et al. studied the association between the number of teeth lost and the presence of atherosclerosis in the carotid arteries. They found atherosclerotic plaques in the carotid arteries in 46% of those who lost 0 to 9 teeth and 60% of those who lost more than 10 teeth [40].

Correlations between atherosclerotic lesions in the carotid arteries and the number of missing teeth, plaque index, and DMFT (decayed + missing + filled teeth) were evaluated by Schilinger et al. by performing an ultrasound examination of the carotid arteries. The researchers showed that edentulous subjects had more advanced atherosclerotic lesions in the carotid arteries [41].

Holmlund et al. demonstrated that subjects with fewer than 10 teeth had a sevenfold increased risk of mortality from myocardial infarction than those with more than 25 teeth. Subjects with fewer teeth had more advanced atherosclerotic lesions in the carotid arteries than those with more teeth [42].

Researchers believe that a lack of teeth correlates with the incidence of heart attack and the development of atherosclerosis. A lack of teeth is a marker for a history of periodontal inflammatory disease, which affects the development of atherosclerosis and heart attack; however, the results of our study are different. The number of edentulous patients was not high compared to the results of epidemiological studies in Poland, in which 35.5% of people over 60 years of age are edentulous (according to WHO), and in some provinces, even 46.6% [43]. Our study showed only 17.5% of edentulous people after myocardial infarction, with a mean age of 57 years. Unfortunately, the retained dentition was mainly affected by periodontitis among the subjects. The low percentage of edentulous individuals may also reflect a lack of dental reporting and lack of oral hygiene and the presence of periodontitis-affected teeth in the mouth that are eligible for extraction, and a focus on systemic infections. The mean value of periodontal pocket depth was also compared between the results available in the literature. In our study, the mean depth of periodontal pockets (PD = 3.06 mm) was less than the study by Wojcicka-Rubin et al. (PD = 3.27 mm) [37]. A study by Bochniak et al. found that post-MI subjects had a higher mean periodontal pocket depth (PD = 4.04 mm) than non-MI subjects (PD = 3.32 mm) [44]. Similar results were obtained by Cueto et al. [45]. The mean value of periodontal pocket depth in post-infarction patients (PD = 2.61 mm) was higher than those without cardiovascular disease (PD = 2.27 mm).

As outlined, cardiovascular disease and periodontitis can have adverse effects on health; treatment and control of periodontitis alone do not guarantee cardiovascular disease prevention, but can only modify its course.

The study in post-MI patients shows a great need for preventing and treating periodontitis. Therefore, it is necessary to improve patients’ periodontal and dental care after myocardial infarction and the cooperation between cardiology and periodontology specialists. Patients are generally unaware of the relationship between general diseases and oral conditions and often have no way to obtain this information. Kalińska et al. reported that they obtained information on this topic first from dentists, secondly, from the mass media, including the Internet, and only in third place did the information come from the general practitioner [46]. We believe that a routine medical procedure should be imparting basic knowledge about oral health and its impact on public health.

Our study has some limitations. Unfortunately, the most recent classification of periodontal disease was not used in the study. In addition, the study group is not very large because all available patients in a given period were considered. In addition, no study size calculations were performed. We plan to expand the study group in future studies and use the new classification.

## 5. Conclusions

Control of risk factors is significant in preventing and treating periodontal disease and heart disease. In light of the above-described etiopathogenesis of atherosclerotic disease and its connection with periodontal disease, it should be emphasized how important the prevention and treatment of periodontitis is, especially for patients in the so-called high-risk group for cardiovascular disease. Dentists’ introduction of an appropriate prophylactic and therapeutic plan may constitute both primary and secondary prevention of cardiovascular diseases.

Based on the study, the following conclusions were drawn:

1. Results obtained in the study support the thesis that people after myocardial infarction have worse periodontal status than healthy individuals. There is a significant need to prevent and treat periodontitis in patients after myocardial infarction. Therefore, it is necessary to increase periodontal care and cooperation between cardiology and periodontology specialists.
2. It was shown that patients with hypercholesterolemia had more advanced periodontal inflammation expressed by CPI and CAL values than those without hypercholesterolemia and lower total cholesterol levels correlate with less periodontitis based on these indicators. CAL values were statistically higher in the group of subjects with high TG levels. It was shown that subjects with more advanced periodontitis had a higher number of atherosclerosis-affected coronary vessels, which confirms the negative impact of periodontitis on general health. Men were more likely to have both myocardial infarction and severe periodontitis, indicating that gender is a risk factor for these conditions.
3. It was shown that periodontal inflammation was more advanced in subjects with 1 to 6 teeth, as confirmed by the periodontal status parameters PD and CAL. It can be presumed that this small number of retained teeth was overloaded because it acted like a complete dentition. The results obtained testify to the poor periodontal status of the subjects.

## Figures and Tables

**Table 1:** CPITN index according to Ainamo et al.

CPI Code	TN Category
0	No treatment needs
1	I
2	II
3	II
4	III

The categories of treatment needs were as follows: TN I: only oral hygiene instruction is required; TN II: TN I + professional treatment for removal of dental deposits (scaling) is required; TN III: TNI+ TNII + comprehensive periodontal treatment is required.

**Table 2:** Characteristics of the study population concerning periodontal pocket (PD) depth at interproximal sites by gender.

PD (mm)	Male	%	Female	%	Σ	
	*n*		*n*		*n*	%
≤3 mm	3	2.83%	2	7.69%	5	3.78%
4–5 mm	42	39.62%	13	50.00%	55	41.67%
≥6 mm	61	57.55%	11	42.31%	72	54.55%
Σ	106		26		132	
Chi^2^ Pearson	2.73		df = 2		*p* = 0.25518	
R rank Spearman	−0.13		t = −1.528		*p* = 0.12886	

**Table 3:** Characteristics of the study population concerning connective tissue attachment loss (CAL) values at interproximal sites by gender.

CAL	Male	%	Female	%	Σ	
	*n*		*n*		*n*	%
1–2 mm	7	6.60%	0	0.00%	7	5.30%
3–4 mm	9	8.49%	2	7.69%	11	8.33%
≥5 mm	90	84.91%	24	92.31%	114	86.37%
Σ	106		26		132	
Chi^2^ Pearson	1.87		df = 2		*p* = 0.39349	
R rank Spearman	0.09		t = 1.0469		*p* = 0.29708	

**Table 4:** Division of periodontitis according to Page and Eke classification in the study population after myocardial infarction.

	Male	%	Female	%	Σ	
	*n*		*n*		*n*	%
None or mild inflammation	8	7.55%	5	19.23%	13	9.85%
Moderate inflammation	36	33.96%	12	46.15%	48	36.37%
Severe inflammation	62	58.49%	9	34.62%	71	53.78%
Σ	106		26		132	
Chi^2^ Pearson	5.96		df = 2		*p* = 0.05080	
R rank Spearman	−0.21		t = −2.424		*p* = 0.01672	

**Table 5:** Periodontal status of the study population as assessed by the CPITN index.

Code CPI	Number of Patients	%	Code TN	Number of Patients	%
0	0	0.00%	No treatment	0	0.00%
1	0	0.00%	I	0	0.00%
2	3	2.28%	II	55	41.67%
3	52	39.39%			
4	77	58.33%	III	77	58.33%

**Table 6:** Risk factors for ACS.

Risk Factor		Female		Male		Σ		*p*
		*n*	%	*n*	%	*n*	%	
Stress	Yes	25	71.43%	66	53.66%	91	57.59%	*p* = 0.06053
	No	10	28.57%	57	46.34%	67	42.41%	
Genetic history	Yes	10	30.30%	30	24.59%	40	25.81%	*p* = 0.50580
	No	23	69.70%	92	75.41%	115	74. 19%	
hypertension	Yes	28	80.00%	101	80.80%	129	80.63%	*p* = 0.91571
	No	7	20.00%	24	19.20%	31	19.37%	
diabetes	yes	13	37.14%	32	25.60%	45	28.13%	*p* = 0.17944
	no	22	62.86%	93	74.40%	115	61.87%	
Nicotinism	Never	9	25.71%	23	18.40%	32	20.00%	*p* = 0.33898
	Former	4	11.43%	28	22.40%	32	20.00%	
	Current	22	62.86%	74	59.20%	96	60.00%	
BMI	Correct	11	31.43%	21	16.80%	32	20.00%	*p* = 0.15501
	Overweight	13	37.14%	53	42.40%	66	41.25%	
	Obesity	11	31.43%	51	40.80%	62	38.75%	
WHR	Gynoid type	2	5.71%	46	37.10%	48	30.19%	*p* = 0.00036
	Android type	33	94.29%	78	62.90%	111	69.81%	

**Table 7:** Lipidogram, CRP, leukocytes, ejection fraction, number of coronary lesions by gender. Abbreviations: CRP, c-reactive protein; FW, ejection fraction; LDL; low-density lipoprotein; HDL, high-density lipoprotein; TG, triglycerides.

		Female		Male		Σ		*p*
		*n*	%	*n*	%	*n*	%	
CRP	<=5 mg/L	13	38.24%	53	42.74%	66	41.77%	*p* = 0.63691
	>5 mg/L	21	61.76%	71	57.26%	92	58.23%	
leucocytes	4–10 G/L	11	33.33%	53	45.69%	64	35.57%	*p* = 0.20579
	>10 G/L	22	66.67%	63	54.31%	85	64.43%	
FW	≤35%	3	8.57%	19	15.20%	22	13.75%	*p* = 0.45909
	36–49%	11	31.43%	44	35.20%	55	34.37%	
	50–75%	21	60.00%	62	49.60%	83	51.87%	
Number of altered coronary vessels	1–2	10	28.57%	38	30.40%	48	30.00%	*p* = 0.83472
	3–4	25	71.43%	87	69.60%	112	70.00%	
dyslipidemia	yes	29	82.86%	97	78.23%	126	79.24%	*p* = 0.55368
	no	6	17.14%	27	21.77%	33	20.76%	
Total cholesterol	≥200	21	60.00%	67	54.03%	88	55.35%	*p* = 0.53055
	<200	14	40.00%	57	45.97%	71	44.65%	
LDL	135	17	48.57%	64	51.61%	81	50.95%	*p* = 0.75059
		18	51.43%	60	48.39%	78	49.05%	
HDL	M < 35, K < 40	10	28.57%	26	20.97%	36	22.64%	*p* = 0.34251
	M ≥ 35, K ≥ 40	25	71.43%	98	79.03%	123	77.36%	
TG	≥150	15	42.86%	60	48.78%	75	46.45%	*p* = 0.53580
	<150	20	57.14%	63	51.22%	83	53.55%	

**Table 8:** The number of teeth and prevalence of diabetes, hypertension, dyslipidemia, and abdominal obesity in patients after acute myocardial infarction.

		Number of Teeth						Chi^2^ Pearson	R Rank Spearman
		0		1–6		>6			
diabetes	no	21	75.00%	16	66.67%	78	72.22%	*p* = 0.79304	*p* = 0.99271
	yes	7	25.00%	8	33.33%	30	27.78%		
	Σ	28	100%	24	100%	108	100%		
hypertension	no	11	32.29%	2	8.33%	18	16.67%	*p* = 0.00871	*p* = 0.07755
	yes	17	60.71%	22	91.67%	90	83.33%		
	Σ	28	100%	24	100%	108	100%		
dyslipidemia	no	9	33.33%	1	4.17%	23	21.30%	*p* = 0.03630	*p* = 0.80540
	yes	18	66.67%	23	95.83%	85	78.70%		
		28	100%	24	100%	108	100%		
WHR	Gynoid type	6	21.43%	5	21.74%	37	34.26%	*p* = 0.26610	*p* = 0.11054
	Android type	22	78.57%	18	78.26%	71	65.74%		

**Table 9:** Hypertension versus mean number of teeth.

	Normal Blood Pressure				Hypertension				ANOVA	Mann–Whitney U
	*n*	Mean	SD	Median	*n*	Mean	SD	Median	*p*	*p*
Numder of teeth	20	18.95	7.58	20.50	112	14.25	7.69	14.00	0.0128	0.0109
BOP	20	0.32	0.24	0.25	112	0.43	0.26	0.38	0.0711	0.0480

**Table 10:** The number of atherosclerotic altered arteries versus mean CAL at interproximal sites.

	1–2				3–4				ANOVA	Mann–Whitney U
	*n*	Mean	SD	Median	*n*	Mean	SD	Median	*p*	*p*
CAL	38	5.95	2.79	6.00	94	6.91	2.25	7.00	0.0390	0.0510

## Data Availability

Data available on request.
